# Organ-Chip Models: Opportunities for Precision Medicine in Pancreatic Cancer

**DOI:** 10.3390/cancers13174487

**Published:** 2021-09-06

**Authors:** Muhammad R. Haque, Trevor H. Rempert, Taslim A. Al-Hilal, Chengyao Wang, Abhinav Bhushan, Faraz Bishehsari

**Affiliations:** 1 Division of Digestive Diseases, Rush Center for Integrated Microbiome & Chronobiology Research, Rush University Medical Center, Chicago, IL 60612, USA; muhammad_r_haque@rush.edu; 2 Department of Biomedical Engineering, Northwestern University, Evanston, IL 60208, USA; trevorrempert2020@u.northwestern.edu; 3 Department of Pharmaceutical Sciences, School of Pharmacy, University of Texas at El Paso, El Paso, TX 79902, USA; taalhilal@utep.edu; 4 Department of Biomedical Engineering, Illinois Institute of Technology, Chicago, IL 60616, USA; cwang108@hawk.iit.edu (C.W.); abhushan@iit.edu (A.B.)

**Keywords:** pancreatic ductal adenocarcinoma, microfluidics, organ-on-a-chip, tumor microenvironment, tissue engineering

## Abstract

**Simple Summary:**

Among all types of cancer, Pancreatic Ductal Adenocarcinoma (PDAC) has one of the lowest survival rates, partly due to the failure of current chemotherapeutics. This treatment failure can be attributed to the complicated nature of the tumor microenvironment, where the rich fibro-inflammatory responses can hinder drug delivery and efficacy at the tumor site. Moreover, the high molecular variations in PDAC create a large heterogeneity in the tumor microenvironment among patients. Current in vivo and in vitro options for drug testing are mostly ineffective in recapitulating the complex cellular interactions and individual variations in the PDAC tumor microenvironment, and as a result, they fail to provide appropriate models for individualized drug screening. Organ-on-a-chip technology combined with patient-derived organoids may provide the opportunity for developing personalized treatment options in PDAC.

**Abstract:**

Pancreatic Ductal Adenocarcinoma (PDAC) is an expeditiously fatal malignancy with a five-year survival rate of 6–8%. Conventional chemotherapeutics fail in many cases due to inadequate primary response and rapidly developing resistance. This treatment failure is particularly challenging in pancreatic cancer because of the high molecular heterogeneity across tumors. Additionally, a rich fibro-inflammatory component within the tumor microenvironment (TME) limits the delivery and effectiveness of anticancer drugs, further contributing to the lack of response or developing resistance to conventional approaches in this cancer. As a result, there is an urgent need to model pancreatic cancer ex vivo to discover effective drug regimens, including those targeting the components of the TME on an individualized basis. Patient-derived three-dimensional (3D) organoid technology has provided a unique opportunity to study patient-specific cancerous epithelium. Patient-derived organoids cultured with the TME components can more accurately reflect the in vivo tumor environment. Here we present the advances in organoid technology and multicellular platforms that could allow for the development of “organ-on-a-chip” approaches to recapitulate the complex cellular interactions in PDAC tumors. We highlight the current advances of the organ-on-a-chip-based cancer models and discuss their potential for the preclinical selection of individualized treatment in PDAC.

## 1. Introduction

Pancreatic ductal adenocarcinoma (PDAC) accounts for approximately 90% of pancreatic cancers and has one of the worst prognosis and survival rates in cancer overall [1]. Developing reliable preclinical platforms to test novel treatments could improve translating knowledge from bench to bedside [2]. Cancer cell lines have been the mainstay of preclinical in vitro cancer research for decades. Cell lines have been fundamental in gaining knowledge in cancer biology and are widely used in laboratories due to their lower cost and convenience in handling. However, their predictability of in vivo drug response is limited by several factors, including their adherent monolayer growth, clonality (homogenous population of cells), and lack of interaction with other cell types [3,4]. This is particularly important in PDAC, where the tumor microenvironment (TME) plays an important role in pathogenesis and drug response. Laboratory animals provide us with superior cancer models that better mimic the complexity of human cancers. However, the translatability of tumor drug response from animal models to humans has been hampered partly due to the lack of human tumor heterogeneity in the cellular level and the interspecies differences in tumor immunity, contributing to the failure of otherwise successful animal-based targeted therapies in clinical trials [5,6]. Additionally, high cost and long turnaround time call for alternative approaches [7].

Unlike cell lines or animal models of PDAC, human pancreatic tumors are highly heterogeneous. A subset of patients who may biologically benefit from a targeted drug may not represent significant numbers in a trial to drive clinical effectiveness [8]. Therefore, an individualized preclinical platform based on a PDAC patient’s tumor-specific profile could advance treatments from the one-size-fits-all approach to precision oncology in PDAC.

In recent years, advancements in our ability to derive 3D-cultured primary cells in the form of organoids from the patient’s tissue have moved us closer to individualized medicine in cancer [9,10]. Tissue-derived adult stem cells cultured in 3D hydrogel can self-organize themselves into organotypic structures of organoids [2,11]. However, organoids mainly contain cancerous epithelial cells without the interface with the other components of the TME [11,12]. TME in PDAC is characterized by infiltration of several types of stromal and immune cells resulting in a strong desmoplastic reaction through active and bidirectional crosstalk between cancerous epithelium and surrounding cell types [13]. The TME contributes to the development and progression of the tumor and has an established role in drug resistance [14,15].

A number of in vitro models have been developed to address the limitation of the lack of tumor extracellular matrix (ECM) in the conventional models of cancer and drug screening platforms [11]. In this regard, microfluidic chips are cutting-edge devices that process fluids in micro-sized channels [16] and allow the culture of multiple cell types within a matrix—so-called ‘organ-on-a-chip (OOC)’ technology [17,18,19]. OOC allows us to recapitulate 3D multicellular architecture and microengineering of TME with the potential to bridge the gaps between bench and bedside by providing screening platforms for testing anticancer agents before reaching human clinical trials [11]. 

This review outlines the potential of OOC technology in modeling cancer, particularly in PDAC and its microenvironment. We introduce recent developments and discuss how the current OOC models, combined with organoid technology, could provide insight into PDAC mechanisms and improved cancer therapeutics.

## 2. Organ-on-a-Chip (OOC) Technology in Cancer

Microfluidic chip devices are preferably fabricated on transparent surfaces such as glass or transparent polymer to make them amenable for microscopic imaging [11,20]. It is desirable for chip devices to be disposable. This makes the use of polymers attractive because of their safe and eco-friendly characteristics [21,22]. Polydimethylsiloxane (PDMS) has gained widespread adoption in the fields of the microfluidic chip, tissue engineering, and cancer biology due to its biocompatibility and ease of fabrication [20,23]. PDMS is oxygen permeable, supporting the culture of cells involved in PDAC TME, thus appropriate for cancer studies [24,25]. A detailed technical review of the materials and design of microfluidic devices is out of the scope of this article and can be found elsewhere [11,20,22,26].

In contrast to static cell culture systems in culture flasks and plates, a chip enables the modeling of in vivo physical conditions by allowing a controlled flow of culture medium into the cell chambers. The flow rates can be tailored to mimic the shear stress of the respective organ [26,27]. Our ability to mimic such dynamic cues (i.e., mechanical forces, hypoxia, and matrix stiffness) on a chip is crucial in modeling cellular events in cancer. For example, epithelial to mesenchymal transition (EMT), a key event in cancer progression and invasion, responds to such dynamic forces in the tumor [28,29,30,31]. The phenotypic transition of the cancerous epithelium into mesenchymal faith enhances their pro-survival tone, migratory capacity, and metastasis [30,32]. Dynamic laminar microfluidic platforms could show how flow-based shear stress promotes EMT in cancer [33,34]. Lung tumoroids culture in such flow-based microfluidic chips express higher EMT markers compared with tumoroids in static conditions [28]. Thus, the maneuverable features of microfluidic platforms allow us to evaluate the impact of physical stressors such as changes in the flow and shear stress on the mechanism of cancer progression.

The culture of several cell types within such a dynamic system has made microfluidic chips a desirable in vitro platform towards building more complex organ mimics [23,35,36]. Integration of additional devices and biosensors in the platform has further advanced its translational application for on-chip analysis. For instance, dielectrophoresis-type devices or dynamic-ELISA on a chip allow the measurement of secreted proteins and molecules in real-time [35,36,37,38,39,40]. Together, microfluidic devices incorporating multiple cell types in a physiologically relevant microenvironment with physical, biochemical, and optical sensing capabilities could be instrumental in better modeling molecular and/or cellular characterizations of cancer biology towards an individualized OOC platform for ex vivo drug testing in cancer [41]. This is important, as the TME, including vasculature, immune cells, and non-cellular components surrounding the cancerous epithelium, play a major role in tumor growth and drug resistance [42]. Here we will briefly list some of the crucial cellular processes occurring in the TME that have been successfully recapitulated in microfluidic devices.

### 2.1. Interaction of Cancerous Epithelium with Cellular Components of Tumor Microenvironment

When modeling a specific tumor on a chip, a desired goal is to include cancer cells among the other major cell types typically present in that TME [43]. Compared to traditional well-plate inserts, microfluidic channels provide adequate spatial organization and compartmentalization for culturing tumor spheroids and organoids with other cell types in vitro, where the interaction of the individual’s primary cancer cells could be studied with other components of the TME [44]. The co-culture of 3D tumor spheroids with cancer-associated fibroblasts (CAFs) within hydrogel scaffold on-chip has shown to be useful in studying cell–cell interactions [45]. In this study, the growth of human colorectal carcinoma cell spheroids was increased when co-cultured with fibroblasts. The co-culture enhanced fibroblast activation and migration, suggested bidirectional crosstalk between the cancer cells and the fibroblasts in the TME. When metastatic breast cancer cells were cultured with tumor-associated macrophages within a microfluidic channel, tumor-associated macrophages invaded areas containing the cancer cells [46]. In a pre-cancerous OOC model, co-culture of mammary epithelial cells with human mammary fibroblasts promoted normal ductal carcinoma transition to an invasive phenotype [47]. 

### 2.2. Angiogenesis

Tumor growth and metastasis are dependent on the formation of new blood vessels for vascular support [48]. Tumor secreted factors help vascular network formation, distinct from normal vasculature due to structural abnormalities, disorganized layout, increased leakiness, and aberrant osmotic forces [49,50,51,52]. 

The leaky tumor vasculature was shown to occur after co-culturing human ovarian cancer spheroid and endothelial cells within a dense matrix on a chip [53]. This platform predicted nanoparticle accumulation in the in vivo tumor model and provided a powerful tool for evaluating nanoparticle delivery to the tumor cells. Modeling a microvascular network on a 3D microfluidic system recapitulated in vivo histologic and biochemical features of lung and brain cancers and provided a versatile platform for testing the efficacy of anti-angiogenic drugs [54,55]. Similar approaches in hematologic malignancies were able to assess anti-angiogenic agents in individual patients [56,57]. More recently, in a breast OOC cancer model, breast cancer-driven organoids loaded into a multi-chamber microfluidic chip supported the 3D growth of angiogenic blood vessels towards the cancerous organoids [58]. By showing a reduction in tumor growth with paclitaxel’s vascular perfusion, this model also confirmed the potential use of the organoid-based device in personalized drug response ex-vivo. Together, these studies propose microfluidic chips as promising platforms for modeling angiogenesis in cancer and assessing individual responses to anti-angiogenic agents.

### 2.3. Metastasis

Metastasis begins when cancer cells invade the basement membrane and migrate through the tumor matrix into lymphatics or blood vessels to reach a remote site [11,59,60,61]. Different mechanical properties of each space, such as confinement and stiffness, can affect this migration [62,63,64]. The effect of confinement on cancer cell migration was successfully shown using microfluidic devices. The incorporation of cancer cells into different channels of chip devices showed that confinement alone, in the absence of any chemical gradient, can influence cancer migration [65]. Exposing cells to drugs that alter microtubule dynamics, such as Taxol, seemed to lower the migratory capability.

A large body of evidence exists to show the utility of chip devices in modeling metastasis via cell–cell interaction and cellular signaling [66]. Addition of chemokines (e.g., TNF-α) and immune cells (i.e., macrophages) to a chip device induced vascular leakiness and intravasation of tumor cells to the endothelial layer as quantified by real-time visualization [67]. Similarly, a microfluidic device was successfully employed to model bone metastasis in breast cancer, where extravasation of metastatic breast cancer cells to bone marrow-driven mesenchymal stem cells was observed [68]. 

Overall, these data and similar studies show the utility of microfluidic chips in modeling tumor cells within their microenvironment for better studying tumor characteristics [11,69]. Given the key involvement of the TME in tumor progression and drug response in PDAC, chips seem to be a promising platform to build individualized OOCs for PDAC. Next, we will review the challenges of modeling TME in PDAC and the promises of chip platforms.

## 3. OOCs to Model TME in PDAC

**Figure 1:**
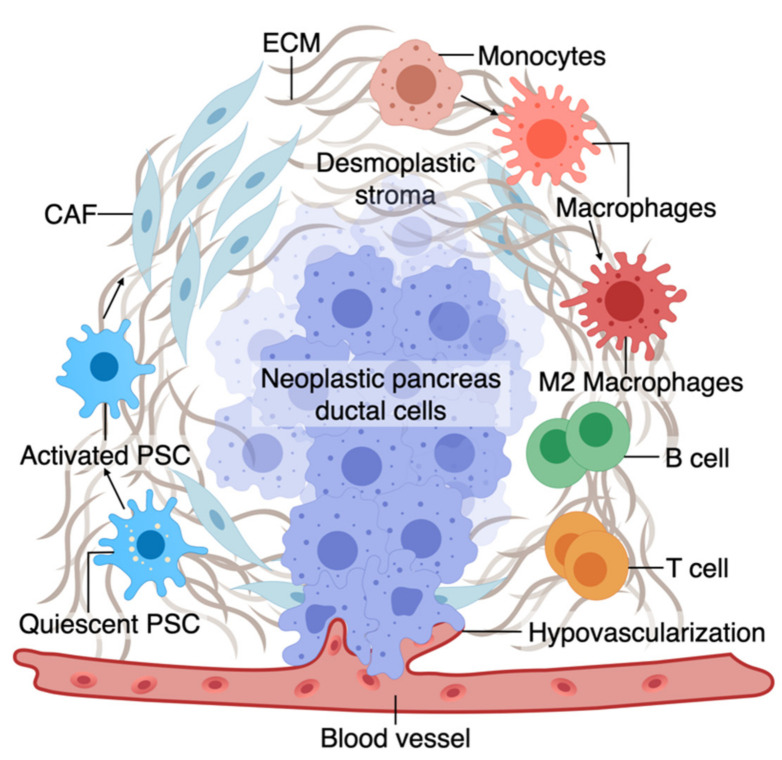
A. The tumor microenvironment of PDAC is composed of interactions among different cell types. At an early stage, interactions of transforming neoplastic ductal cells with pancreatic stellate cells, fibroblasts, and macrophages result in initiating a fibroinflammatory process and subsequent involvement of an adaptive immune response, including recruitment of T cells. As the tumor progresses, cancer cells invade the blood vessels and occupy the vessel lumen, referred to as endothelial ablation. Endothelial ablation and a desmoplastic stroma, accompanied by a suppressive immune environment, reduce drug delivery and chemoresistance at the site.

The TME has an active role in tumor progression, immune evasion, and drug response in PDAC [70,71]. Non-cellular components of TME of pancreas tumors are composed of ECM and dense fibrous tissue, regarded as “desmoplasia” (Figure 1) [72]. The dense ECM is mainly composed of matrix proteins such as collagen secreted by the cellular components of TME such as fibroblasts and pancreatic stellate cells (PSCs) [73]. PSCs can reduce cancer cell death upon chemotherapy induction by releasing soluble factors or activating stemness signaling pathways in cancer cells [15]. Several other immune cell types are present in the TME, among which macrophages are the most abundant that gauge both the innate and adaptive immune responses against the tumor [73]. Similar to PSCs, macrophages in interaction with cancer cells can shift towards a pro-tumorigenic phenotype, which leads to increased cancer cell stemness and growth [74]. Macrophages are also crucial in rendering chemoresistance to conventional chemotherapeutics for PDAC [75]. Pancreatic epithelial ducts secrete alkali in the epical side and create an alkaline pH in the microenvironment to prevent the breakdown of secreted pre-enzymes in the normal pancreas before reaching the small bowel lumen [76]. In PDAC, dysregulation of ion channel transporters (i.e., Ca^2+^ and K^+^ channels) and the tumor hypovascularization contribute to the acidification of the microenvironment, which further promotes cancerous characteristics in the epithelium (e.g., selection of EMT phenotypes) while shifting the stromal cells (i.e., PSC and macrophages) towards protumorigenic phenotypes [76,77]. While there are numerous other cell types in the TME (e.g., T cells, B cells, Dendritic cells), we focus on collagen-producing cells and macrophages as two major components of the TME in PDAC. In the following sections, we highlight the characteristics and plasticity of these cell types within the tumor area before discussing the opportunities provided by OOCs to model TME in PDAC.

### 3.1. Macrophages and Fibroblasts in PDAC

Macrophages in the pancreatic tissue are immune cells that can arise from the embryonic precursors traced back to the extraembryonic yolk or infiltrating monocytes from myeloid precursors in the bone marrow [78,79,80,81,82,83]. The tissue macrophages can change their function (polarization) in response to surrounding signals. While macrophages were traditionally classified into M1 and M2 subtypes, mounting evidence supports the presence of a wide phenotypic spectrum between M1 with stronger killing properties to M2 that can contribute to a smoldering chronic inflammatory state in cancer [83,84,85,86,87]. M1 macrophages, through the production of nitrogen and oxygen derivatives, possess anti-tumorigenic ability by identifying and destroying cancer cells through phagocytosis [84].

In contrast, M2 macrophages can promote tumor growth via multiple mechanisms, as discussed in detail elsewhere [84,88]. While M1 macrophages could be predominant in the TME during the early stages of cancer formation, the M2-type phenotype becomes more abundant as the tumor progresses. In fact, tumor-associated macrophages are more similar to M2-types and predict poor survival [88,89,90]. Macrophage polarization or recruitment within the pancreas tissue occurs via crosstalk with cancerous epithelium and other components of the TME. In line with the growing literature on the crosstalk between macrophages and cancerous epithelium in PDAC, we also showed that early carcinogenesis signaling in the pancreatic epithelium could shift macrophages towards M2-like cells and that polarized macrophages could further promote cancer formation via induction of inflammatory signaling [9,73].

The major source of matrix deposition in the TME is cancer-associated fibroblasts (CAFs) that can arise from the PSCs, the resident mesenchymal cells in the pancreas [91,92,93]. Cancer cells activate resident PSCs, which differentiate into CAFs and secrete matrix proteins such as collagen, contributing to the dense ECM [89]. Activated PSCs also secrete factors to induce tumor growth, progression, and metastasis [90,91,94,95]. 

While the dense ECM has been traditionally considered to help tumor progression, recent studies support a more complex role for ECM’s contribution to PDAC progression, which could be stage and context-dependent [96,97]. However, at a late stage, the ECM contributes to tumor chemoresistance via multiple mechanisms such as cancer cell sensitivity, drug cytotoxicity, and reduced drug delivery [98,99,100]. Tumor fibroblasts are associated with poor drug response and disease survival partly by offsetting chemotherapy-induced apoptosis via soluble factors or activating stemness signaling pathways in the cancer cells [101,102,103,104,105]. Similar to macrophages, pancreatic fibroblasts could be phenotypically plastic and dynamic in response to the surrounding tumor stimuli [106,107]. Although continuous matrix deposition turns on the signaling pathways to boost the malignant phenotype, more desmoplasia contributes to tumor progression and drug resistance [108,109,110]. While the role of stroma in promoting resistance in drug response is well accepted, recent animal studies show conflicting roles of stroma in tumor formation in PDAC. Protective effects of stroma were suggested by increased tumor aggression upon stroma reduction in a mouse model where Sonic hedgehog (Shh), a soluble ligand that drives the formation of desmoplastic stroma, was deleted [111].

### 3.2. TME on Chip Models of PDAC

Recent advancements of PDAC TME on chip models are summarized in Table 1. A biomimetic ductal TME on a chip, where ductal pancreatic cancer epithelium cells were surrounded by collagen matrix in the chip, recapitulated the histopathology of PDAC [112]. The tumor heterogeneity was reconstituted using pancreatic cancer cells from GEMM carrying KRAS, CDKN2A, and TP53 mutations, key driver mutations of human PDAC. This model revealed the complex interactions between cancerous epithelial cells in PDAC, leading them to be more aggressive and invasive [112].

PDAC is highly metastatic even at an early stage and escapes into the blood circulation [113,114]. In a biomimetic OOCs based model of PDAC, endothelial ablation was seen where PDAC cells invaded the matrix toward the endothelial lumen, wrapped around the blood vessel, spread along the length, and finally invaded the vessel and occupied the vessel lumen [66]. This finding of endothelial ablation in the 3D organotypic model was further confirmed in the tumor-bearing mouse models [66].

If we are able to mimic the active TME on multicellular OOCs, can we use these platforms to model PDAC drug response in vitro? A humanized microfluidic device, where PSCs were cultured with PANC-1 cells, represented expected histologic features of PDAC and showed the potential adjuvant therapeutic activity of anti-TME agents to conventional chemotherapies [115]. The data suggest that besides studying cytotoxicity, this model has the potential to determine the effects of TME compactness and collagen reorganization on PDAC therapeutics [115]. A similar drug response to Cisplatin has been shown in a microfluidic chamber cultured with different PDAC cells in ECM-enriched environments [116]. 

Moving towards a patient-based ex vivo preclinical platform, work is in progress to use tissue-driven cells in OCC models of cancer. In this regard, organoids have made it possible to test drugs on the individual’s tumor cells in the lab. Next, we will discuss how the integration of organotypic technologies in OOCs could allow us to model complex cellular interactions, and the therapeutic activity of anticancer drugs, with the potential to design novel therapeutics at the individual level. 

## 4. Individualized PDAC Model on Chip

The variation in sensitivity to anticancer drugs among different patients highlights the requirement for more precise treatment selection [117]. We and others have shown that organoids retain a high degree of similarity to the original tissue, including PDAC [9,10]. PDOs are proposed to provide an opportunity for a personalized in vitro platform to test drug sensitivity in individual patients [118]. In PDAC, organoid technology is instrumental in optimizing the use of sparse tissue collected from clinically indicated endoscopic fine needle biopsies (FNBs) performed for tissue diagnosis. This circumvents the particular challenges to precision medicine in PDAC, which stems from limited access to surgically-naïve specimens for pre-treatment screening (>80% of PDACs are unresectable) and rapid patient deterioration [119]. While organoids are superior to conventional cells for predicting drug response, they often show uncertain growth and considerable heterogeneity and are challenging to manipulate using conventional in vitro techniques [117]. Therefore, culturing them in microfluidic OOC platforms that mimic 3D tissue architecture and better facilitate nutrient and gas exchange could be more faithful in modeling the disease [120]. Work is in progress to make the organoid-based models more complex to simulate in vivo tissue structures [2,121].

Such a personalized in vitro chip model was recently developed using PDOs derived from PDAC tumor biopsy, fibroblasts, and endothelial cells tri-cultured in a perfusable 96-well based OOC system [25]. Symbiotic interaction between the PDOs and fibroblasts was observed with an elevated proliferation and increased PDO diameter in the co-culture system. Moreover, fibroblast contributed to chemoresistance to gemcitabine by secreting collagen, which added to the matrix stiffness and acted as a physical barrier to drug delivery. This co-culture platform showed the importance of the relationship between patient cells and desmoplastic ECM and provided a better understanding of chemotherapeutic agents’ bioavailability inside vascularized tumor tissues. Such a model could demonstrate a particular anticancer drug’s sensitivity to an individual patient in the lab before applying in the clinic. 

## 5. Challenges and Future Directions

Studying the interactions between cancerous epithelium and non-epithelial cells of the TME is challenging as it requires monitoring the disease over time in a living system. Since OOCs can recapitulate the biomechanical and biochemical microenvironments of in vivo tissues, OOC models can be used for studying the complex nature of PDAC. Furthermore, OOC technology can help understand additional cellular interactions in PDAC, including interactions between cancer and perivascular cells surrounding and supporting blood vessels. The ultimate goal of microfluidic OOC devices is to create a complex multicellular model of human pancreatic cancer on a chip for drug testing and therapeutics. PDAC is typically characterized by vascular collapse, hypoxia, and increased stiffness due to excessive dense matrix, all of which contribute to poor drug response. The future optimization of OOC devices will allow the incorporation of multiple biomechanical and biochemical factors, such as blood flow, stiffness, high interstitial pressure, and hypoxic microenvironment that influences the PDAC progression, drug delivery, and immunotherapy. OOC devices themselves do not require attending to the ethical concerns associated with animal testing, and their ability to be generated from specific patient cells can render them the superior method of individualized drug testing.

PDAC organoid culture also remains under development, and one of the most pressing issues in organoid technology is the variability of the system, which started with the isolation of patient samples leading to the establishment of organoids and their cryopreservation. To address the issue of reproducibility, two independent labs can run basic tests in the same system for functionality and toxicity. The effect of the ECM and immune infiltrates from donors or patients on PDAC OOC culture is yet to be defined. The heterogeneity in the composition of the immune cells of PDAC can heavily influence the outcomes of the model itself, let alone affect the chemical or genetic screening. This obstacle should be overcome by incorporating patient immune cells into the OOC culture system. The use of PDOs is also limited by directly exposing them to the test molecules instead of delivering them through a nearby lumen. Thus, to better recapitulate the disease, integrating PDAC architecture, hallmarks of the PDAC desmoplastic TME, and vascular transport are necessary. Consideration should be given to creating a personalized PDAC model using the PDO along with the patient’s cells, such as monocyte/macrophages, cancer-associated fibroblasts, or endothelial cells, and incorporating into the OOC system.

## 6. Conclusions

Compared to other tumor types, pancreatic tumors are especially rich in fibroinflammatory responses that limit chemotherapy access to the tumor. The response is driven by the tumor microenvironment and affects both the pathophysiology of cancer progression and the drug response. Single-layer platforms that only use cancerous epithelial cells may not sufficiently represent the in vivo drug response. Current animal models also fail to recreate the human PDAC TME to test drugs adequately. Although the OOC platform is a unique way to avoid flaws related to the preclinical studies on animal models, it is important to make this platform appropriate for a specific purpose as they are not generic. There is no existing OOC model yet to replace animal models as a whole, and more research is needed on this platform to identify possible therapeutic failures which are not detected in preclinical animal models. Still, as an alternative source, OOC technology helps generate independent data sets in different stages of the drug discovery pipeline, which can be used to obtain precise information on a drug’s effect prior to clinical trials. Results from PDO-based OOC in conjunction with clinical data could ultimately guide the way to individualized-based drug discovery. Such models have significant value in translational cancer research, especially in pancreatic cancers where current treatment options are limited. However, the issue of standardization is still a limitation of the OOC approach. 

## Figures and Tables

**Table 1:** Published articles on PDAC on chips.

Author, Publication Year	Cells Used	Model Name	Potential Applicaiton	Predictive Value
Lai Benjamin et al., 2020 [25]	HUVEC, human dermal fibroblasts (FBs), patient-derived organoids from PDAC	Vascularized human PDAC on InVADE platform	The co-culture model shows that cancer organoids and fibroblasts exert a synergistic effect on cancer progression. This model has the potential to demonstrate the inhibitory effects of chemotherapeutic drugs in vitro in the presence of desmoplasia.	The vascularized human PDAC model can capture the hallmarks of an evolving TME. Such a model helps make personalized PDAC on-chip to observe drug responses before applying to the patients in the clinic.
Bradney et al., 2020 [112]	Murine pancreatic cancer cell line from KPC genotype with *KRAS* and *TP53* mutations, and KIC genotype with *KRAS* and *CDKN2A* mutation	Ductal tumor-microenvironment-on-chip (dT-MOC)	Recapitulates the anatomical hallmark of PDAC, where a collagen matrix surrounds ductal cancer cells. Therefore, this model is suitable to study intratumoral heterogeneity.	GEMMs are time-consuming to breed. Thus, such biomimetic dT-MOC composed of cancer cells from GEMM are desired to understand the complex TME in a shorter period.
Nguyen et al., 2019 [66]	HUVEC, primary mouse pancreatic cancer cell PD7591	PDAC-on-a-chip with a biomimetic blood vessel	Explains that hypovascularity in PDAC may be caused by endothelial ablation, and ALK7 signaling contributes to this phenomenon.	This 3D organotypic model provides the platform to understand complex cellular interaction with mechanistic insights.
Beer et al., 2017 [116]	BxPC3, PANC-1, MIA PaCa-2	3D cultured PDAC cell lines in HepaChip^®^ microfluidic chamber	Provides a cell culture environment suitable for maintaining cell viability, morphology, and growth similar to 3D culture.	Such a platform has the potential to be used in improved diagnosis and prognosis of PDAC. Also, personalized pharmacological drug testing can be performed on such devices.
Drifka et al., 2013 [115]	Human primary PSC, PANC-1 PDAC cells	3D microfluidic in vitro model of PDAC	This pathophysiologically relevant human PDAC model provides an alternative platform to evaluate the preclinical efficacy of therapeutic candidates.	The ability of this model to accommodate real-time observation helps us understand the interaction of ECM components with cancer cells within the PDAC TME. Moreover, by incorporating primary human cells, this model has the complexity of human PDAC TME. This property can be utilized to test drug efficacy in vitro before reaching the clinic.

## Abbreviations

CAF	cancer-associated fibroblast
TME	tumor microenvironment
ECM	extracellular matrix
PDAC	pancreatic ductal adenocarcinoma
OOC	organ-on-a-chip
PDMS	polydimethylsiloxane
EMT	epithelial to mesenchymal transition
